# Surgical Resection of Ruptured Fibrolamellar Hepatocellular Carcinoma

**DOI:** 10.1155/2013/679565

**Published:** 2013-07-18

**Authors:** Vincenzo Minutolo, Alessio Licciardello, Manuel Arena, Orazio Minutolo, Raffaele Lanteri, Goffredo Arena

**Affiliations:** ^1^Department of Surgical Sciences, Organ Transplantation and Advanced Technologies, University of Catania, Via Santa Sofia 84, 95123 Catania, Italy; ^2^Department of Surgery, Royal Victoria Hospital, McGill University, 687 Pine Avenue, Montreal, QC, Canada H3A1A1

## Abstract

Fibrolamellar hepatocellular carcinoma (FLH) is a rare primary tumor of the liver, which typically arises from noncirrhotic livers and affects patients below the age of 35. We report on a 29-year-old male patient who presented with a ruptured FLH and was treated with surgical resection. Options for treatment and review of the management are described.

## 1. Introduction

Fibrolamellar hepatocellular carcinoma (FLC) is a rare primary liver tumor presenting earlier in life than nonfibrolamellar hepatocellular carcinoma [1]. Ruptured hepatocellular carcinoma (HCC) occurs in 3% to 15% of patients with HCC. The mortality in the acute phase is very high and stands at around 25%–75% of cases. We report the first case, to our knowledge, of ruptured FLC, which was treated with surgical resection.

## 2. Case Report

**Figure 1:**
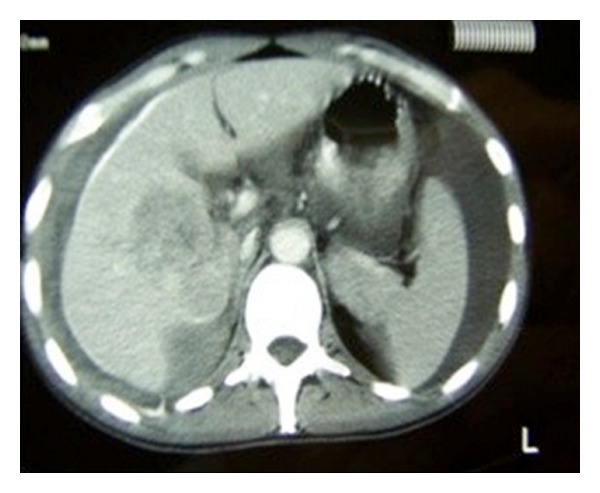
CT abdomen showing the liver mass and hemoperitoneum.

**Figure 2:**
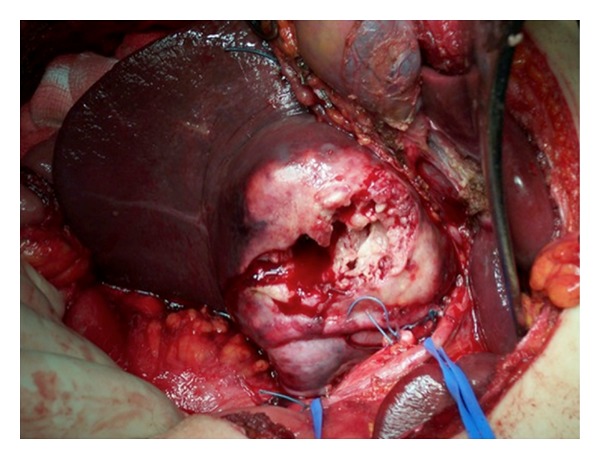
Intraoperative image of the FLC invading the hepatoduodenal ligament and abutting the duodenum.

**Figure 3:**
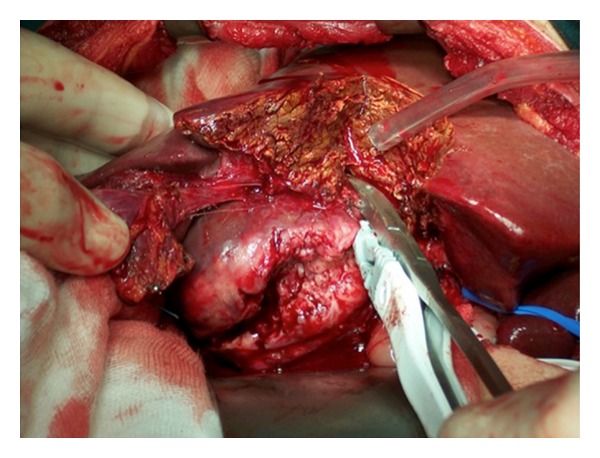
Resection of segments V and VI with electrosurgical device.

**Figure 4:**
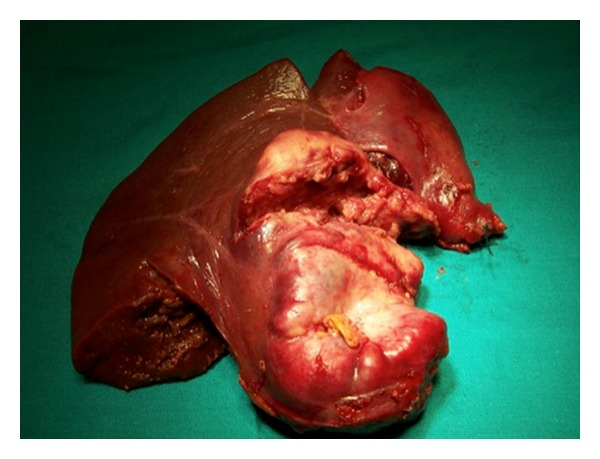
Segments V and VI en bloc with gallbladder.

 A 29-year-old male patient came to the Emergency Department complaining of a sudden onset of right upper quadrant pain, which was associated with nausea and vomiting. On history the patient reported some chronic pain in the right upper quadrant, which had been present in the previous days, which suddenly had become worse prior to admission to the hospital. Past medical history was completely unremarkable. On clinical examination the patient appeared pale and in distress. Blood pressure was 80/60 mm Hg, heart rate was 120, and respiratory rate was 40. The abdomen was painful with guarding and rebound in all four quadrants. Laboratory tests showed Hb of 69 g/dL and WBC 15.000 *μ*L. A FAST ultrasound performed in emergency showed an eight by five cm lesion in segments V and VI of the liver, with diffuse hemoperitoneum. An emergency abdominal CT scan was requested, and it showed evidence of an eight by five cm ruptured hepatic lesion with massive hemoperitoneum and leakage of contrast medium. The lesion had a big extrahepatic component with possible invasion of the hepatoduodenal ligament and gallbladder (Figure 1). While the CT scan was being performed the patient became clinically unstable, and therefore decision was taken to bring the patient to the operating room for an emergency surgery. In the operating room, a Chevron incision was performed to gain access into the abdomen. Packing of the liver together with a Pringle maneuver was performed to achieve hepatic inflow occlusion and slow down the bleeding. After aspiration of three liters of blood that had been accumulating in the abdominal cavity we proceeded to a thorough inspection of the bleeding mass in the liver. The mass was invading the hepatoduodenal ligament, and it was extending into but not penetrating the second part of the duodenum (Figure 2). No other visible masses were seen in the liver or in the peritoneal cavity. Resection of segments V and VI of the liver en bloc with the gallbladder was performed using LigaSure device (Figures 3 and 4). The patient was then transferred to ICU. He made an uneventful recovery and was discharged on POD 10. Pathology confirmed the suspicion that the mass was a ruptured fibrolamellar hepatocellular carcinoma with lymphovascular invasion. Follow-up CT scans after 6 months showed recurrence of the disease in the liver bilaterally and peritoneal metastases, which were treated with chemotherapy. The patient died 26 months after the surgery.

## 3. Discussion

Fibrolamellar hepatocellular carcinoma is a rare primary malignant tumor of the liver, which was first described in 1956. Unlike conventional hepatocellular carcinomas, FLC typically affects younger patients, often below the age of 35, without underlying liver disease, and both sexes are affected equally [1]. Microscopically, FLCs look like circumscribed masses characterized by well-differentiated polygonal cells with eosinophilic and granular cytoplasm surrounded by thick, fibrous stroma arranged in bands [2]. FL-HCCs are best diagnosed preoperatively by abdominal CT and MRI imaging. These tumours are usually heterogeneous on CT imaging with areas of hypervascularity. On MRI, tumours are usually T1 hypointense and T2 hyperintense, and the use of a gadolinium contrast agent during MRI results in heterogeneous enhancement [2]. Patients with FLC treated with surgical resection have a more favorable outcome than nonfibrolamellar hepatocellular carcinomas with reported 5-year survival ranging from 58% to 82% [1]. Spontaneous rupture of HCC occurs in 3% to 26% of all patients with HCC, and high mortality rate, in the range of 32% to 67%, has been reported [3, 4] The pathogenesis of liver rupture is not well known; several hypotheses have been proposed in the literature, including a fast-growing tumor resulting in central necrosis, coagulopathy, fragility, vascular congestion and venous compression, or infiltration of the tumor [5]. Tumor size >5 cm and extrahepatic invasion have been associated with high propensity to rupture [6–8]. Clinically the patients present with abdominal pain, tenderness, and peritonitis. Feature of cirrhosis might be present or not. Options for treatment of ruptured HCC are several, and there is no consensus on the most effective [9]. Surgical ligation of the hepatic artery, perihepatic packing, plication, and hepatectomy including wedge hepatectomy, mono- or bisegmentectomy, and lobectomy have been proposed [10]. If the patient's hemodynamical conditions allow, a two-staged approach involving radiologic transarterial embolization (TAE) for hemostasis followed by staged hepatectomy is preferred over emergency hepatectomy [7, 10]. This approach permits to stabilize the patient from a hemodynamical point of view, assess the liver function, and stage the cancer to better plan the surgical resection. Several reports in the literature also demonstrate good outcomes following surgical resection of the bleeding HCC especially in Child A and B patients [6, 9, 11, 12]. The benefits from emergency liver surgery appear more evident in patients with small tumors located in accessible locations and in noncirrhotic livers, as it was the case in our patient. The benefits of this approach include hemostasis and treatment of the cancer in one single stage, decreasing rate of tumor spreading, and prevention of the complications from TAE such as liver abscess, rebleeding, and implanted peritoneal metastases. Although patients with FLC treated with surgical resection have a more favorable outcome than nonfibrolamellar hepatocellular carcinomas, the significant spillage of tumor secondary to the rupture promoted the dissemination of the cancer with subsequent recurrence of the disease and early demise of the patient.

## 4. Conclusion

Ruptured HCC is a complication burdened by high mortality. Treatment needs to be tailored to the hemodynamic conditions of the patients and to the liver function. TAE followed by staged hepatectomy might be the preferred approach in hemodynamic stable patients with advanced liver disease. Surgery should be the ideal treatment in patients with no underlying liver disease or preserved liver function.
